# Synthesis of novel (*E*)-1-(2-(2-(4(dimethylamino) benzylidene) hydrazinyl)-4-methylthiazol-5-yl)ethanone derivatives as ecto-5′-nucleotidase inhibitors

**DOI:** 10.1098/rsos.180837

**Published:** 2018-09-12

**Authors:** Sidra Hassan, Pervaiz Ali Channar, Fayaz Ali Larik, Aamer Saeed, Hamid Saeed Shah, Joanna Lecka, Jean Sévigny, Jamshed Iqbal

**Affiliations:** 1 Centre for Advanced Drug Research, COMSATS University Islamabad, Abbottabad Campus, Abbottabad 22060, Pakistan; 2 Department of Chemistry, Quaid-i-Azam University, 45320 Islamabad, Pakistan; 3 Faculty of Pharmacy, University of Sargodha, Sargodha 40100, Pakistan; 4 Département de microbiologie-infectiologie et d′immunologie, Faculté de Médecine, Université Laval, Québec, Canada G1V 0A6; 5 Centre de Recherche du CHU de Québec, Université Laval, Québec, Canada G1V 4G2

**Keywords:** ecto-5′-nucleotidase, thiazole, Schiff base, azomethines

## Abstract

Ecto-5′-nucleotidase (e5′NT), a membrane-bound enzyme and an essential member of ecto-nucleotidases which regulates extracellular purinergic signalling. Their upregulation results in various disease conditions, for example, inflammation, hypoxia and cancer. Therefore, efforts have been made to synthesize potent and selective inhibitors of e5′NT. Here we have synthesized, characterized and evaluated six thiazole derivatives **(3a–3f)** as potent e5′NT inhibitors. Among all derivatives, the compound (*E*)-1-(4-methyl-2-(2-(pyridin-3-ylmethylene)hydrazinyl) thiazol-5-yl)ethanone **(3a)** exhibited maximum inhibition towards both human and rat enzymes. However, their potency against *h*-e5′NT was 24-fold higher than *r*-e5′NT. Only two compounds exhibited inhibitory behaviour towards *r*-e5′NT. The molecular structures of these derivatives were confirmed with the help of solid-state characterization through NMR (^1^H and ^13^C), FTIR and elemental analysis. Additionally, molecular docking was also implemented to explain putative bonding interaction between the active site of an enzyme and potent inhibitors.

## 1. Background

As a regulator of adenosine signalling pathway, the membrane-bound ecto-5′-nucleotidase (e5′NT, CD73) speeds up the final reaction step that involves the hydrolysis of extracellular nucleotides and their conversion from adenosine monophosphate (AMP) to adenosine [1]. These enzymes belong to metallophosphoesterase superfamily which contains divalent cation in its active site [2]. The end product of this catalytic activity is adenosine molecule which further activates P1 receptor and accelerates a number of physiological and pathophysiological processes like anti-inflammatory, immunosuppressive, tranquillizing, vasodilatation and antidiuretic effects [3]. It is evident from previous reports that numerous cancer cells display high levels of e5′NT, which ultimately increases the intensity of adenosine production to promote angiogenesis and T-cells death [4]. An e5′NT has been involved in cancer progression by performing several functions; e.g. enzymatic and non-enzymatic functions. An uncontrolled enzymatic activity results in stimulation of adenosine receptors that reflects its role in breast cancer cells migration as well as invasion and adhesion to the extracellular membrane (ECM). Irrespective of its catalytic activity, an e5′NT functions as co-receptor in T-cell activation or promotes cell interaction with ECM component as well as migration and regulates cell–cell adhesion [5,6]. An e5′NT has been supposed to be a motility factor in the development and progression of cancers. Hence several studies have suggested the correlation between e5′NT expression and tumour progression [7]. Therefore, the e5′NT enzyme is considered a significant target in cancer therapy. Eukaryotic e5′NTs are competitively inhibited by ADP and ATP, regarded as physiological inhibitors. Previously, small inhibitory molecules or antibodies have been investigated that resulted into reduction of metastasis and tumour growth [8]. It has been found that ADP analogues such as AMPCP resulted in inhibition of all forms of e5′NT. In addition, several other molecules like anthraquinone, sulfonamide and flavonoid-based compounds were found to be active against e5′NT [9,10]. All these known molecules lack specificity and selectivity towards their target. Hence the therapeutic importance of e5′NT plus need of potent and selective moieties encourages us to synthesize a series of compounds as a potential therapeutic agent in various disease conditions.

Azomethines are widely known as Schiff bases; which represent a pharmacologically active class of organic compounds. Schiff bases have grabbed immense importance in medicinal chemistry research due to their widespread therapeutic activities including anti-microbial [11,12], anti-tumour [13], anti-mycobacterial [14], trypanocidal [15], anti-inflammatory [16], anti-HIV [17], anti-diabetic [18] and anti-malarial activities [19]. Apart from medicinal significance, Schiff bases elicit applications in dyes and pigments, catalysts, polymer stabilizers and serve as intermediates in organic synthesis. Azomethines can be easily synthesized by the condensation reaction of amines with suitable aldehydes or ketones.

Combination of two or more bioactive nuclei is considered as a clever approach in drug designing. Hence, we tagged Schiff bases with thiazole ring. Like azomethines, thiazole ring has been recognized as medicinal active moiety and has displayed numerous biological activities such as antibacterial [20], anti-inflammatory [21], anti-fungal [22], anti-hypertensive [23], anti-malarial [24], anti-HIV [25], anti-convulsant [26], anti-tumour [27], herbicidal, insecticidal, anti-schistosomiasis and anthelmintic [28]. Several drugs such as anti-HIV (Ritonavir) [25], anti-inflammatory (Fanetizole and Meloxicam) [26,28], anti-ulcer (Nizatidine) [29] and insecticide (Imidacloprid) possess a thiazole ring, which further cements the legacy of thiazole as medicinally privileged structure.

## 2. Material and methods

All materials were used without further purification. The COS-7 cells were obtained from ATCC USA. Lipofectamine, transfecting reagent, DMEM/F-12, *h*-e5′NT, *r*-e5′NT AMP, CaCl_2_, Tris–HCl and MgCl_2_ were purchased from Sigma-Aldrich Germany.

### 2.1. Synthesis of 1-(2-hydrazinyl-4-methylthiazol-5-yl)ethanone(2)

A 0.02 M of 3-chloropentane-2,4-dione (**1**) in dry distilled methanol (50 ml) was refluxed for 7 h in the presence of thiosemicarbazide (0.02 M). The intermediates formed during each step of reaction were evaluated with the help of thin layer chromatograph (TLC). When the reaction was completed the mixture was decanted in ice cold water. The acquired precipitates (86% yield) were filtered and recrystallized in ethanol.

### 2.2. Common method for production of (*E*)-1-(2-(2-(4-(dimethylamino)benzylidene) hydrazinyl)-4-methylthiazol-5-yl)ethanones (**3a**–**f**)

**Figure 1.**
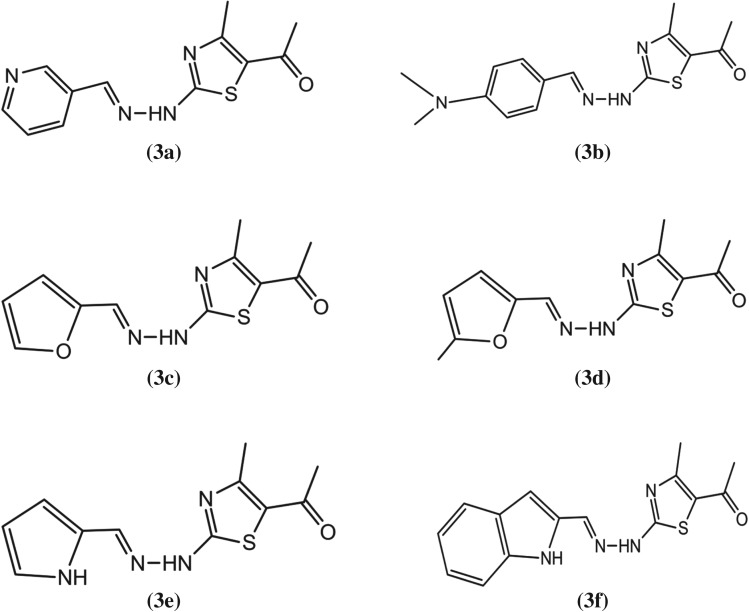
Chemical structures of following test compounds. (*E*)-1-(4-methyl-2-(2-(pyridin-3-ylmethylene)hydrazinyl)thiazol-5-yl)ethanone [**3a**], (*E*)-1-(2-(2-(4-(dimethylamino) benzylidene)hydrazinyl)-4-methylthiazol-5-yl)ethanone [**3b**], (*E*)-1-(2-(2-(furan-2-ylmethylene)hydrazinyl)-4-methylthiazol-5-yl)ethanone [**3c**], (*E*)-1-(4-methyl-2-(2-((5-methylfuran-2-yl)methylene)hydrazinyl)thiazol-5-yl)ethanone [**3d**], (*E*)-1-(2-(2-((1*H*-pyrrol-2-yl)methylene)hydrazinyl)-4-methylthiazol-5-yl)ethanone [**3e**], (*E*)-1-(2-(2-((1*H*-indol-2-yl)methylene)hydrazinyl)-4-methylthiazol-5-yl)ethanone [**3f**].

A 3.65 mmol sample of 1-(2-hydrazinyl-4-methylthiazol-5-yl)ethanone (**2**) was dissolved in 15 ml of absolute alcohol followed by mixing of aldehyde (3.70 mmol) under continuous stirring and mild heating. The reaction mixture was refluxed with uninterrupted stirring for 10–12 h and reaction mixture was quenched with acetic acid to obtain **3a–f**. The completion of the reaction was confirmed by the help of TLC having solvent mixture of petroleum ether and ethyl acetate in ratio of 8 : 2. The reaction mixture was maintained in a cold environment and strained on Buckner funnel then washed with cold ethanol. The yield and melting point were noted and recrystallization was performed with ethanol (figure 1).

### 2.3. Biochemical assays

#### 2.3.1. Cell transfection with ecto-5′-nucleotidase

The plasmids expressing e5′NT, either rat or human, were used to transfect the COS-7 cells through lipofectamine [30]. The confluent cells were allowed to incubate in DMEM/F-12 having plasmid DNA (6 µg) and transfecting reagent (24 µl) for 5 h at ambient temperature (37°C). To discontinue transfection, a specified volume of DMEM/F-12 was added, containing FBS (20%). These cells were then harvested after 2–3 days.

#### 2.3.2. Production of membrane fractions

The transfected cells were separated from harvesting buffer (45 mM Tris buffer, 95 mM NaCl, and 0.1 mM PMSF, pH 7.5) through scraping, followed by washing with Tris buffer and then allowed to centrifuge by spinning at 300 r.p.m. at 4°C for 5 min [30]. Finally, the cells remained suspended in harvesting buffer solution containing aprotinin (10 µg ml^−1^). As a result of sonication, the produced cellular debris was removed by cold centrifugation operated at 300 r.p.m. for 10 min. Glycerol (7.5%) was mixed with the resultant supernatant. The Bradford microplate assay was employed for estimation of protein concentration where albumin was used as a standard [31].

#### 2.3.3. Ecto-5′-nucleotidase inhibition assay

The e5′NT inhibition assay was performed with respect to our previously described procedure [32] on P/ACE MDQ capillary electrophoresis system (Beckman Instruments, Fullerton, CA, USA) provided with UV detection system. The samples were prepared in the assay buffer (1 mM CaCl_2_, 10 mM Tris HCl and 2 mM MgCl_2_, pH 7.4) and analysed at 0.1 mM concentration. The total assay volume (100 µl) comprises sample (10 µl), 10 µl of *h*-e5′NT (6.94 µg ml^−1^) protein extract or *r*-e5′NT (7.17 µg ml^−1^) and 70 µl of assay buffer. The above mixture was allowed to incubate at 37°C for 10 min. Subsequently, biochemical reaction was started with the addition of substrate (10 µl) and AMP (500 µM). The mixture was incubated again for 30 min at 37°C. The enzyme-substrate reaction was stopped by thermal denaturation for the duration of 20 min by placing the mixture in a water bath at 99°C. Then, 50 µl of obtained mixture was filled into CE mini vials and injected with 0.5 psi into the capillary in 5 s, whereas 15 kV was set to split-up substrate and product peaks. The demonstration of greater than 50% inhibitory activity by compounds on either human or rat enzyme was further investigated for estimation of IC_50_ values. Therefore, successive dilution of each active compound was made and different inhibitor concentrations were assayed to obtain the dose–response curve against both enzymes. All experiments were conducted thrice. The nonlinear regression analysis program (PRISM 5.0) was employed to calculate IC_50_ value.

### 2.4. Molecular docking studies

Molecular docking analysis of every compound was performed to explore binding mode with the active site of *h*-e5′NT as well as *r*-e5′NT target enzymes. X-ray crystallographic structures were present in RCSB Protein Data Bank which was downloaded in the form of PDB ID: 4H2I [33], while X-ray crystallographic structure of *r*-e5′NT was not reported till now, so a previous homology model was selected for docking studies [34]. The two-dimensional structures of all compounds T-1 to T-6 were created via Marvin-ChemAxon suit [35] and transformed into a three-dimensional structure with the Molecular Builder program executed in Molecular Operating Environment (MOE 2014.0901) [36]. Crystal structure of *h*-e5′NT also contained unwanted water along with co-crystallized ligands within binding sites which were removed before docking calculation. Prior to docking analysis, target enzymes along with test compounds were protonated and their energies were reduced up to 0.05 gradient using MMFF94x force field using Protonate three-dimensional tool of MOE 2014.0901. Molecular docking calculations of selected compounds were performed via MOE-Dock tool in MOE 2014.0901 [36]. For molecular docking calculation against *h*-e5′NT, active site was selected nearby co-crystallized ligands. Whereas an *r*-e5′NT Site-Finder utility of MOE was used. Selected compounds were docked into enzyme binding site with the help of Triangular Matching docking protocol. For each ligand-protein complex, 30 conformations were established based on binding free energies. Furthermore, the poses were sorted out on the basis of lowest binding energy values which were regarded as the most stable one with the highest affinity for interaction with receptor. Discovery studio visualizer 4.0 was used for investigating putative binding interaction with side chains of amino acid residues in active pockets of target enzymes [37].

## 3. Results and discussion

### 3.1. Characterization data

The ^**1**^**H NMR** and ^**13**^**C NMR** spectra of few representative compounds are provided in the electronic supplementary material.

#### 3.1.1. (*E*)-1-(4-methyl-2-(2-(pyridin-3-ylmethylene)hydrazinyl)thiazol-5-yl)ethanone (**3a**)

Yellow solid; yield: 78%; R_f_: 0.54; m.p: 251°C; **IR**: 2948 (C–H), 1698(C=O), 1672(C=N),^**1**^**H NMR** (DMSO*-d*_6_, 300 MHz); *δ* = 12.00 (NH), 8.88 (d, *J* = 1.2 Hz, 1H), 8.61 (dd, *J* = 5.0, 1.2 Hz, 1H), 8.46 (s, 1H, HC=N-), 8.11 (dt, *J* = 7.9, 1.3 Hz, 1H), 7.48 (dd, *J* = 8.0, 5.0 Hz, 1H), 2.49 (s, 3H), 2.29 (s, 3H).^**13**^**C NMR** (DMSO, 75 MHz); 174.1, 167.0, 162.0, 154.0, 151.6, 149.6, 140.4, 134.4, 130.5, 124.4, 29.5, 23.7. **Anal. Calcd.** For C_12_H_12_N_4_OS: C, 55.37; H, 4.65; N, 21.52; S, 12.32 found: C, 55.35; H, 4.67; N, 21.50; S, 12.34 found: 260.

#### 3.1.2. (*E*)-1-(2-(2-(4-(dimethylamino) benzylidene)hydrazinyl)-4-methylthiazol-5-yl)ethanone (**3b**)

Brown solid; yield: 78%; R_f_: 0.54; m.p: 252°C; **IR**: 2946 (C–H), 1692(C=O), 1674(C=N),^**1**^**H NMR** (DMSO*-d*_6_, 300 MHz); *δ*= 12.16 (NH), 8.47 (s, 1H, HC=N–), 7.58 (s, 2H, H-o), 7.32 (m, 2H, H-m), 3.02 (s, 6H, 2x CH_3_), 2.02 (s, 3H, CH_3_). 1.92 (s, 3H, CH_3_), **^13^C NMR** (DMSO, 75 MHz); 165.4, 162.1, 150.7, 149.9, 148.4, 131.0, 130.0, 122.0, 116.5, 46.0, 22.0, 16.0. **Anal. Calcd**. For C_15_H_18_N_4_OS: C, 59.58; H, 6.00; S, 10.60 found: C, 59.56; H, 6.02; S, 10.64 found: 302.

#### 3.1.3. (*E*)-1-(2-(2-(furan-2-ylmethylene)hydrazinyl)-4-methylthiazol-5-yl)ethanone (**3c**)

Pink solid; yield: 79%; R_f_: 0.56; m.p: 242°C **IR**: 2932 (C–H), 1683(C=O), 1674(C=N),^**1**^**H NMR** (DMSO*-d*_6_, 300 MHz); *δ* = 12.40 (s NH), 7.82 (d, *J* = 1.2 Hz, 1H), 6.80 (dd, *J* = 5.0, 1.2 Hz, 1 H), 6.67 (dd, 1 H), 7.90 (s, 1 H), 2.40 (s, 3 H), 2.35 (s, 3 H).^**13**^**C NMR** (DMSO, 75 MHz); 188.2, 174.1, 166.1, 156.5, 154.1, 148.0, 146.1, 113.6, 112.1 29.4, 13.0. **Anal. Calcd.** For C_11_H_11_N_3_O_2_S : C, 53.00; H, 4.45; N, 16.86;; S, 12.86 found C, 53.02; H, 4.43; N, 16.84; S, 12.88 found: 249.

#### 3.1.4. (*E*)-1-(4-methyl-2-(2-((5-methylfuran-2-yl)methylene)hydrazinyl)thiazol-5-yl)ethanone (**3d**)

Orange solid; yield: 79%; R_f_: 0.56; m.p: 252°CC; **IR**: 2932 (C–H), 1683(C=O), 1674(C=N),^**1**^**H NMR** (DMSO*-d*_6_, 300 MHz); *δ* = 11.94 (s NH), 8.46 (s, 1H, HC = N), 7.39 (dd, *J* = 21.8, 7.5 Hz, 1H)7.29 (t, *J* = 7.4 Hz, 1H), 2.10 (s, 3H, 2xCH_3_), 1.70 (s, 3H, CH_3_), **^13^C NMR** (DMSO, 75 MHz); 174.4, 165.7, 150.2, 141.1, 132.7 131.9, 122.7, 116.0, 106.0, 28.5, 22.5, 17.5. **Anal. Calcd.** For C_12_H_13_N_3_O_2_S: C, 54.74; H, 4.98; N, 15.96; S, 12.18 found C, 54.742; H, 4.99; N, 15.98; S, 12.17 found: 263.

#### 3.1.5. (*E*)-1-(2-(2-((1*H*-pyrrol-2-yl)methylene)hydrazinyl)-4-methylthiazol-5-yl)ethanone (**3e**)

Black solid; yield: 75%: m.p 235°C R*_f_* : 0.42; **IR;** 3260 (N-H), 3125 (sp^2^CH), 1603 (C=N), 1587 (Ar-C=C), **^1^HNMR**; (300 MHz, DMSO): *δ* 12.88 (s, 1H, NH), 11.12 (s, 1H, NH), 8.36(s, 1H, CH=N), 7.94–6.66 (m, 3H), 2.19 (s, 6H, 2xCH_3_) **^13^C NMR** (DMSO, 75 MHz); 166.3, 166.1, 160.3, 149.3, 148.0 147.0, 143.2, 117.7, 114.7, 24.9, 17.9, **Anal. Calcd.** For C_11_H_12_N_4_OS: C, 53.21; H, 4.87; N, 22.56; S, 12.91 found C, 53.20; H, 4.85; N, 22.58; S, 12.92 found: 248.

#### 3.1.6. (*E*)-1-(2-(2-((1*H*-indol-2-yl)methylene)hydrazinyl)-4-methylthiazol-5-yl)ethanone (**3f**)

**Scheme 1.**
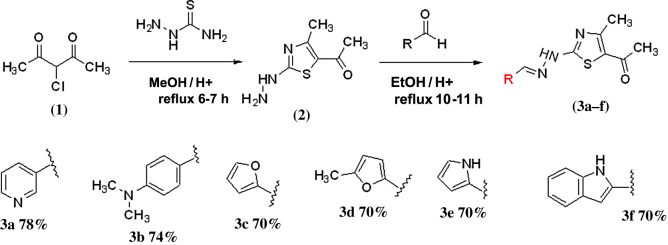
Synthesis mechanism of (*E*)-1-(2-(2-(4-(dimethylamino)benzylidene) hydrazinyl)-4-methylthiazol-5-yl)ethanones (**3a**–**f**).

Brown solid; yield: 79%; R_f_: 0.48; m.p: 272 ^o^C; **IR:** 2912 (C–H), 1673(C=O), 1672(C=N), **^1^HNMR;** (300 MHz, CDCl_3_): *δ* 10.92 (s, 1H, NH), *δ* 10.12 (s, 1H, NH), 7.53 (s, 1H, CH=N),*δ* 7.61 (dd, *J* = 7.5, 1.4 Hz, 1H), 8.58–7.24 (m, 6H), 8.26–7.24 (m, 5H), 7.57 (td, *J* = 7.5, 1.5 Hz, 1H), 7.45–7.38 (m, 2H), 7.33–7.24 (m, 2H), 8.07–5.91 (m, 7H), 7.24–3.49 (m, 2H), 6.77–3.49 (m, 2H). 2.42 (s, 3H, –CH_3_); 2.10 (s, 3H, –CH_3_).**^13^C NMR** (DMSO, 75 MHz); 192.8, 172.5, 160.5, 145.3, 134.5, 133.5, 128.4, 123.5, 122.5, 120.3, 119.5, 112.5 102.5, 26.4. 16.5, **Anal. Calcd.** For C_15_H_14_N_4_OS: C, 60.38; H, 4.73; N, 18.78; S, 10.75 found C, 60.37; H, 4.74; N, 18.80; S, 10.73 found: 298 (scheme 1).

### 3.2. Structure–activity relationship

A series of (*E*)-1-(2-(2-ethylidenehydrazinyl)-4-methylthiazol-5-yl)ethanones was synthesized containing Schiff bases along with thiazole ring. These six derivatives possessing different substitution were tested against e5′NT to evaluate their inhibitory potential. From table 1 data, it was evident that these derivatives had showed higher inhibitory effects towards human enzyme as compared to rat. Except two derivatives, all four derivatives showed inhibition in the range of 0.32 ± 0.03–6.19 ± 0.32 (µM). The compound **3a** and **3b** exhibited maximum inhibition against *h*-e5′NT with IC_50_ ± s.e.m. values 0.32 ± 0.03 and 0.56 ± 0.07 µM, respectively. The compound **3a** exhibited 132-fold higher inhibition against *h*-e5′NT when compared with positive control, i.e. sulfamic acid, with IC_50_ ± s.e.m. value 42.1 ± 7.8 µM. A detailed analysis of compound exhibited the electrophilic behaviour of substituent. As pyridine ring undergoes electrophilic substitution reaction and readily reacts with electronegative molecule, this effect might be responsible for maximum inhibitory potential of compound **3a**. However in the case of compound **3b**, the substitution of dimethylaniline group instead of pyridine on Schiff base could have resulted in stable compound with less reactivity towards its receptor site. It exhibited comparable but less inhibitory activity towards *h*-e5′NT (IC_50_ ± s.e.m.= 0.56 ± 0.07 µM). After initial screening, compounds **3c** and **3d** showed no inhibitory potential toward e5′NT (*h-, r-*) enzymes (table 1) due to presence of furan and 2-methyl furan ring, respectively. Two derivatives (**3e** and **3f**) displayed selective inhibitory behaviour against *h*-e5′NT while these were inactive against *r*-e5′NT with per cent values 10.8 ± 4.25 and 32.7 ± 1.62 (%), respectively. The compound **3e** possessed pyrrole ring substitution at Schiff base that favours its selective reactivity towards *h*-e5′NT. In the case of compound **3f**, the presence of indole ring resulted in selective and significant activity. With regard to *r*-e5′NT, only two derivatives (**3a** and **3b**) displayed some inhibitory activity but were less reactive when compared with their activity against *h*-e5′NT. Both compounds **3a** and **3b** showed their inhibition with IC_50_ ± s.e.m. values 7.81 ± 0.89 and 10.1 ± 0.58 (µM), respectively.

**Table 1.** Ecto-5′-nucleotidase (*h*-e5′NT & *r*-e5′NT) inhibition data (IC_50_ values) for the synthesized compounds. Values are expressed as mean ± s.e.m. of *n* = 3. The IC_50_ is the concentration at which 50% of the enzyme activity is inhibited.

codes	*h*-e5′NT	*r*-e5′NT
	percentage/IC_50_(µM) ± s.e.m.	
**3a**	0.32 ± 0.03	7.81 ± 0.89
**3b**	0.56 ± 0.07	10.1 ± 0.58
**3c**	38.1 ± 5.47%	15.3 ± 2.78%
**3d**	43.6 ± 1.18%	12.3 ± 3.91%
**3e**	3.36 ± 0.12	10.8 ± 4.25%
**3f**	6.19 ± 0.32	32.7 ± 1.62%
sulfamic acid	42.1 ± 7.80	77.3 ± 7.01

### 3.3. Molecular docking analysis

**Figure 2.**
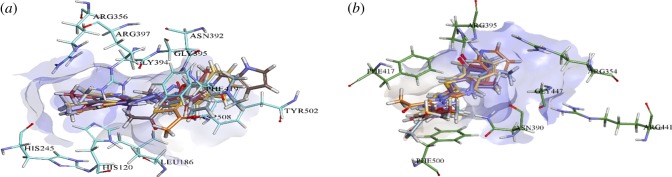
(*a*) Putative binding orientation of all compounds **(3a–3f)** within the active site of *h-*e5′NT. (*b*) Putative binding orientation of all compounds **(3a–3f)** within the active site of *r-*e5′NT.

**Figure 3.**
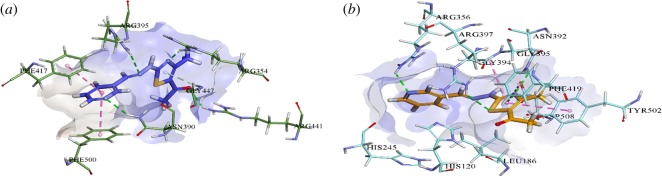
(*a*) Detailed putative binding interactions of potent compound **3a** within the active site of *h-*e5′NT. (*b*) Detailed putative binding interactions of potent compound **3a** within the active site of *r-*e5′NT.

Docking analysis of **3a**–**3f** was executed to investigate putative binding mode with *h*-e5′NT and *r*-e5′NT enzymes. Figure 2*a,b* shows a three-dimensional binding orientation of all docked compounds superimposed within the active site of *h*-e5′NT and *r*-e5′NT, respectively, while figure 3*a,b* demonstrated the binding interaction of **3a** within active pocket of *h*-e5′NT and *r*-e5′NT, respectively. Detailed analysis of binding interactions of **3a** with various amino acid residue of *h*-e5′NT and *r*-e5′NT presented that compound **3a** was making several strong binding interactions such as hydrogen bonding as well as π-π stacked interactions. Binding of **3a** inside active site of *h*-e5′NT was stabilized by four hydrogen bonds as well as two π-π T-shaped stacked interactions as shown in figure 3*a*. The hydrogen bonds were established by **3a** with amino acid residue Arg354, Arg390, Arg395 and Arg441 of *h*-e5′NT enzyme while two π-π bond T-stacked interactions was formed with amino acid residue Phe217 and Phe500 as shown in figure 3*a*. However, the binding of **3a** within the active site of *r*-e5′NT was stabilized by three hydrogen bonds and three π-π T-shaped stacked interactions. Contrary to the binding interface of **3a** inside active site of *h*-e5′NT, three hydrogen bonds formed by **3a** with amino acid residue of *r*-e5′NT involved were Arg356, Asn392 and Arg397 while three π-π stacked interactions were formed by Phe419, Try502 and Gly394.

The compound **3a**, the most potent compound against *h*-e5′NT possesses the pyridine-3-ylmethylene and therefore was found most active. However, the second active compound, **3b**, did not possess the pyrimidin, and instead have dimethylamino group attached to the benzylidene hydrazinyl. The compound showed significant inhibitory potential against both the enzymes, and especially, against *h*-e5′NT.

## 4. Conclusion

In conclusion, a novel series of thiazole derivatives was produced and evaluated for their anti-cancer potential, i.e. e5′NT. The derivatives possessed significant inhibition potential against *h*-e5′NT as compared to *r*-e5′NT. The compound **3a** was found to exhibit maximum inhibition against *h*-e5′NT with IC_50_ value 0.32 ± 0.03 µM that is 24-fold higher than its activity towards *r*-e5′NT. Moreover, molecular docking was also performed to determine their putative binding sites. Hence, these derivatives can be further evaluated for their therapeutic importance in the management of various diseases.

## Supplementary Material

Spectra of (E)–1–(2–(2–(furan–2–ylmethylene)hydrazinyl)–4–methylthiazol–5–yl)ethanone

## Data accessibility

All data are included within the article.
