# Supplementary material for: Synthesis of novel (E)-1-(2-(2-(4(dimethylamino) benzylidene) hydrazinyl)-4-methylthiazol-5-yl)ethanone derivatives as ecto-5′-nucleotidase inhibitors
Source: R Soc Open Sci. 2018 Sep 12;5(9):180837. doi: 10.1098/rsos.180837 (PMC6170555; doi:10.1098/rsos.180837)
Supplement: Spectra of (E)–1–(2–(2–(furan–2–ylmethylene)hydrazinyl)–4–methylthiazol–5–yl)ethanone [file rsos180837supp1.docx]

**Synthesis of novel (*E*)–1–(2–(2–(4(dimethylamino) benzylidene) hydrazinyl) –4–methylthiazol–5–yl)ethanone derivatives as Ecto–5ʹ–Nucleotidase Inhibitors**

Sidra Hassan^1ǂ^, Pervaiz Ali Channar^2ǂ^_,_ Fayaz Ali Larik^2^_,_ Aamer Saeed^^[[1]](#footnote-1)^*2^, Hamid Saeed Shah^1, 3^, Joanna Lecka^4, 5^_,_ Jean Sévigny^4, 5^_,_ Jamshed Iqbal^1^*

*^1^Centre for Advanced Drug Research, COMSATS University Islamabad, Abbottabad Campus, Abbottabad-22060, Pakistan.*

*^2^Department of Chemistry, Quaid–i–Azam University, 45320, Islamabad, Pakistan.*

*^3^Faculty of Pharmacy, University of Sargodha, Sargodha (40100) Pakistan.*

*^4^ Département de microbiologie–infectiologie et d'immunologie, Faculté de Médecine, Université Laval, Québec, QC, G1V 0A6, Canada.*

*^5^ Centre de Recherche du CHU de Québec – Université Laval, Québec, QC, G1V 4G2, Canada.*

^ǂ^ These author contributed equally to the work.

^*^**Corresponding Authors**:

Aamer Saeed; [aamersaeed@yahoo.com](mailto:aamersaeed@yahoo.com),

Jamshed Iqbal; [drjamshed@ciit.net.pk](mailto:drjamshed@ciit.net.pk)

**Address correspondence to:**

Prof. Dr. Jamshed Iqbal

Centre for Advanced Drug Research,

COMSATS University Islamabad, Abbottabad Campus,

Abbottabad, Postal Code 22060, Pakistan

Tel: +92–992–383591–96

Fax: +92–992–383441


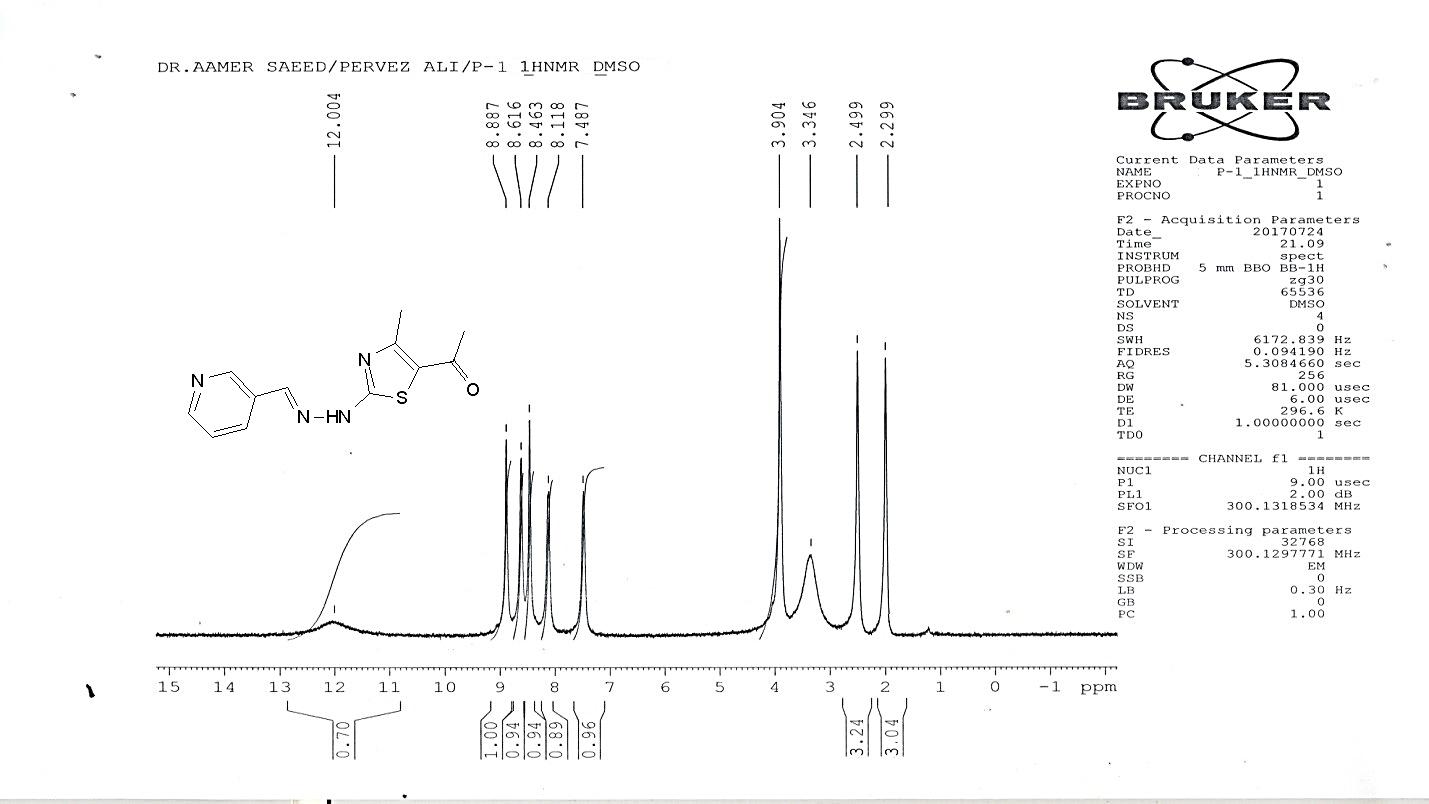


**Figure S1.** The ^1^HNMR spectra of *(E)-*1-(4-methyl-2-(2-(pyridin-3-ylmethylene) hydrazinyl) thiazol-5-yl)ethanone


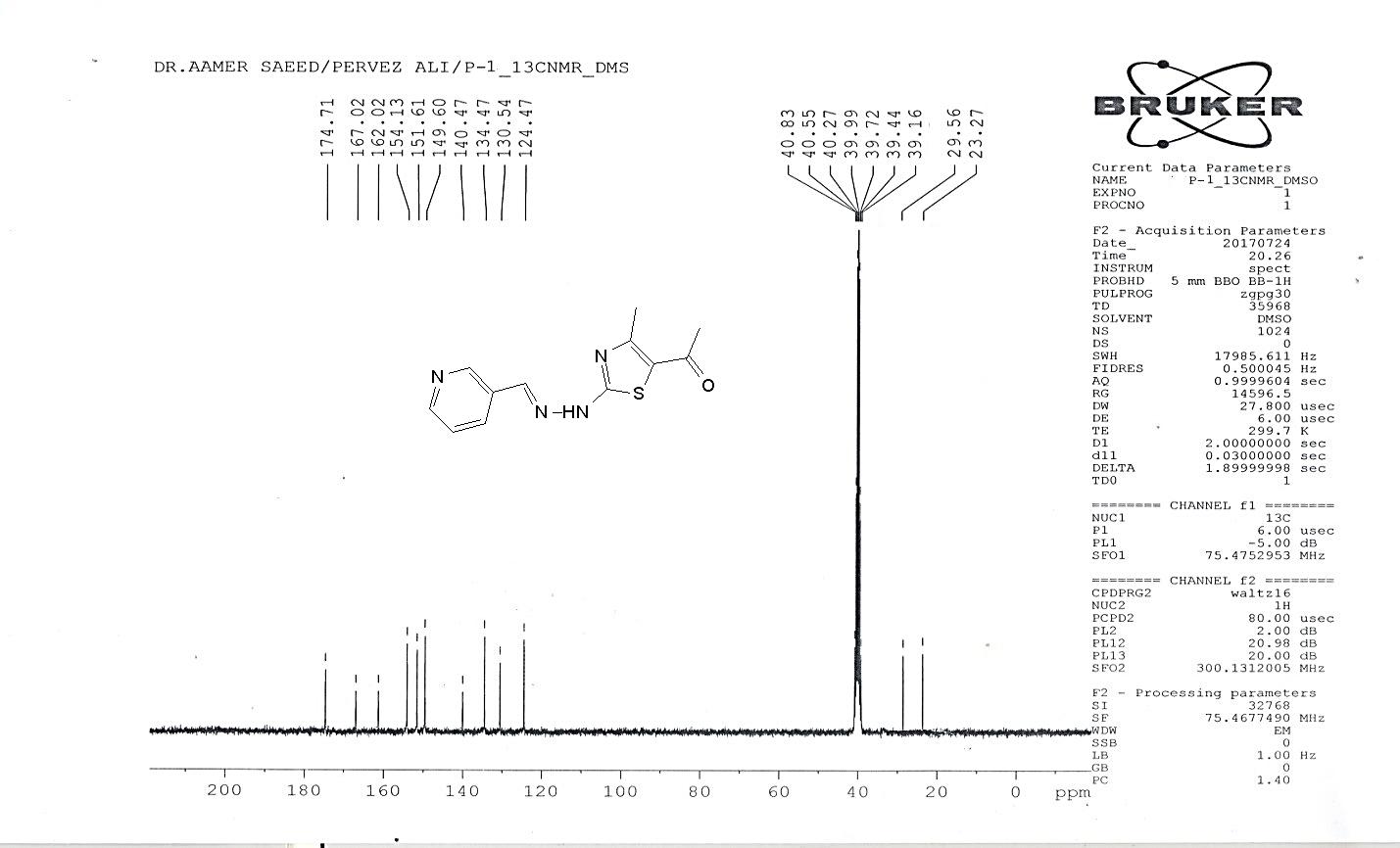


**Figure S2.** The ^13^CNMR spectra of *(E)-*1-(4-methyl-2-(2-(pyridin-3-ylmethylene) hydrazinyl) thiazol-5-yl)ethanone

**
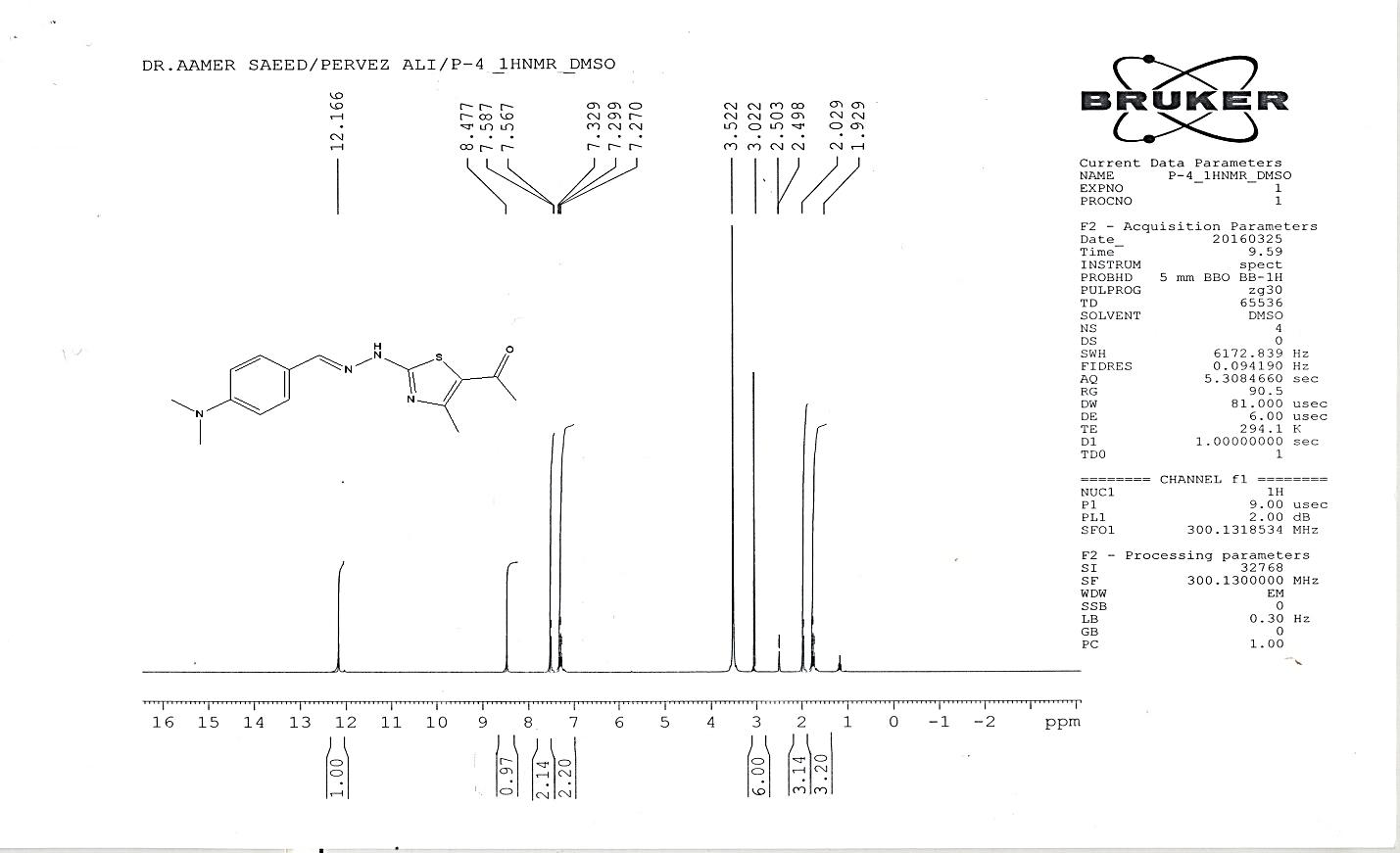
**

**Figure S3.** The ^1^HNMR spectra of *(E)-*1-(2-(2-(4-(dimethylamino) benzylidene)hydrazinyl)-4-methylthiazol-5-yl)ethanone

**
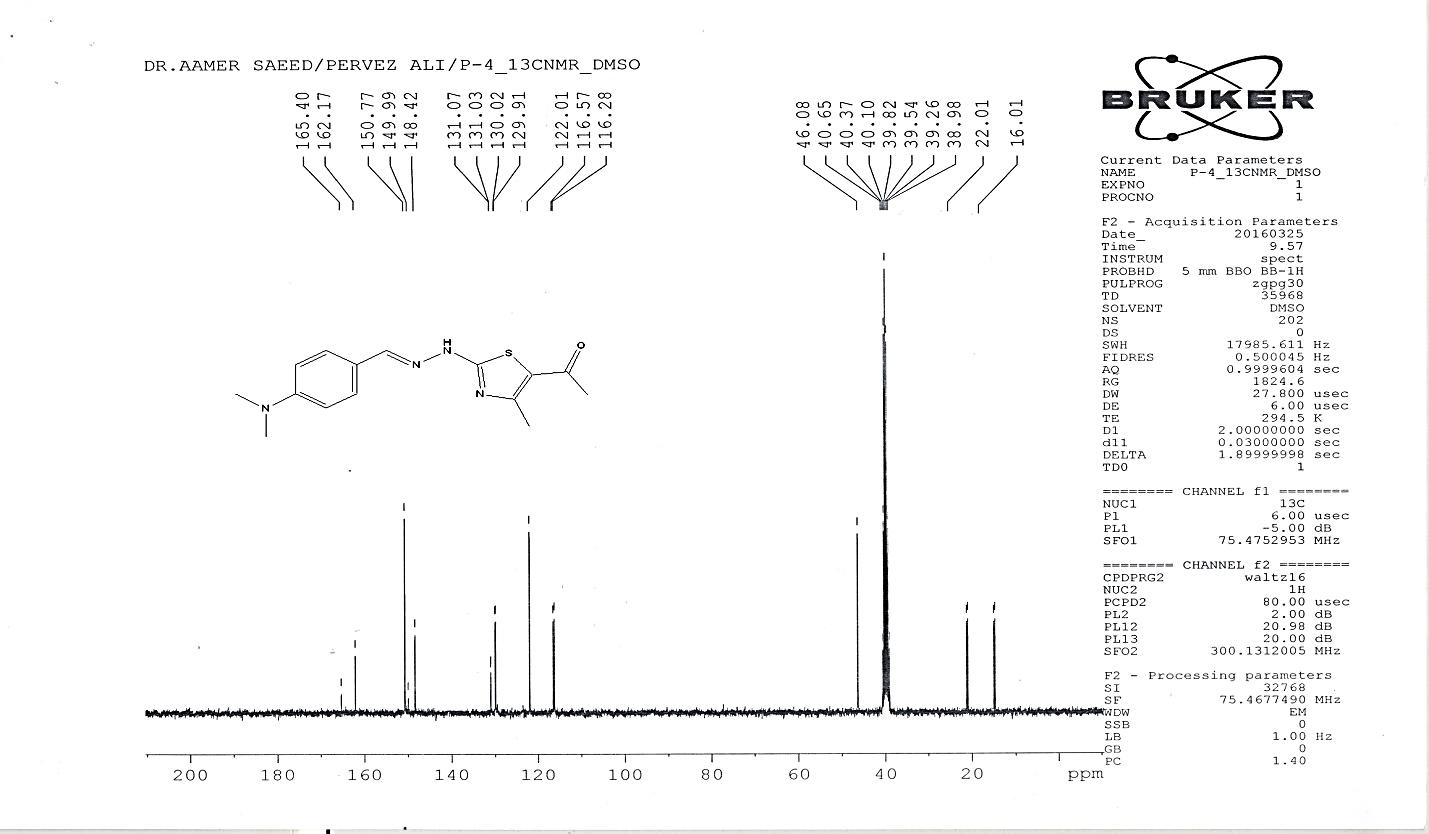
**

**Figure S4.** The ^13^CNMR spectra of *(E)-*1-(2-(2-(4-(dimethylamino) benzylidene)hydrazinyl)-4-methylthiazol-5-yl)ethanone

**Figure S5.** The ^1^HNMR spectra of *(E)-*1-(2-(2-(furan-2-ylmethylene)hydrazinyl)-4-methyl thiazol-5-yl)ethanone


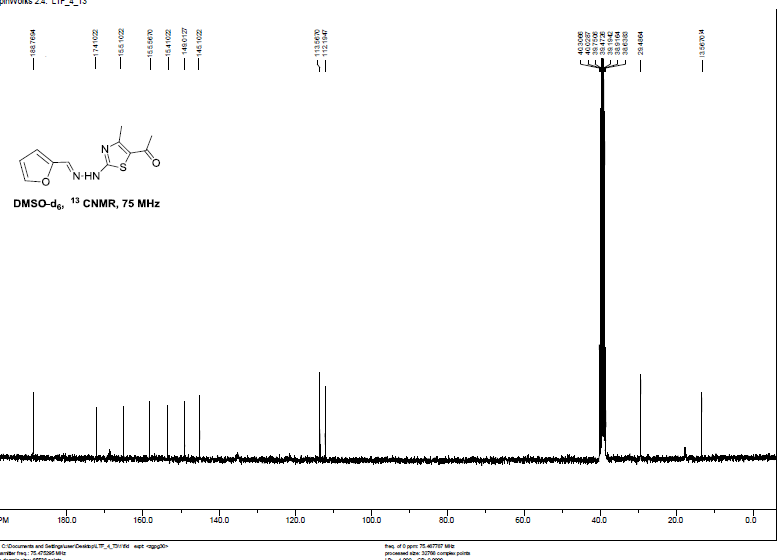


**Figure S6.** The ^13^CNMR spectra of (*E*)–1–(2–(2–(furan–2–ylmethylene)hydrazinyl)–4–methylthiazol–5–yl)ethanone

**
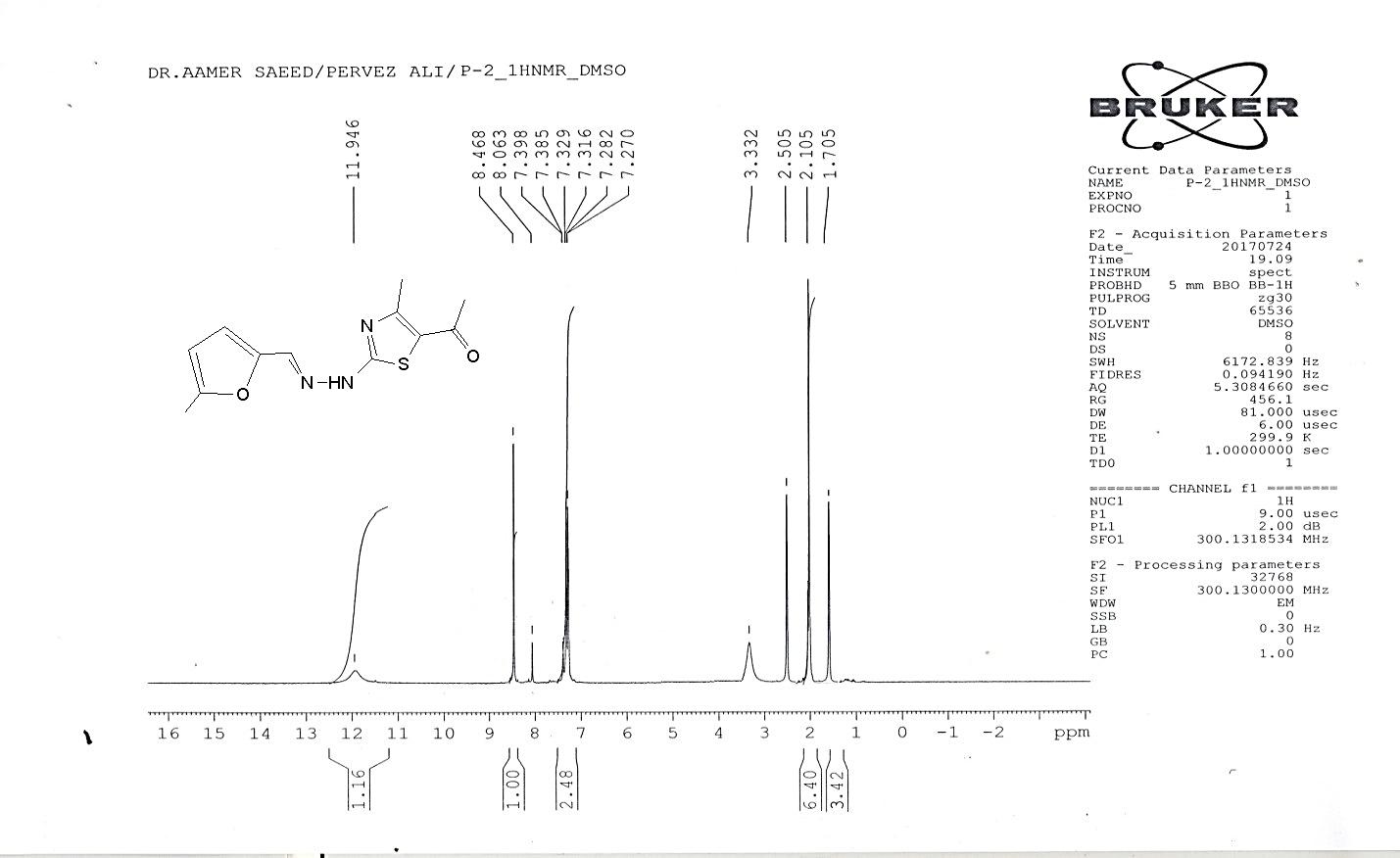
**

**Figure S7.** The ^1^HNMR spectra of *(E)-*1-(4-methyl-2-(2-((5-methylfuran-2-yl) methylene) hydrazinyl)thiazol-5-yl)ethanone

**
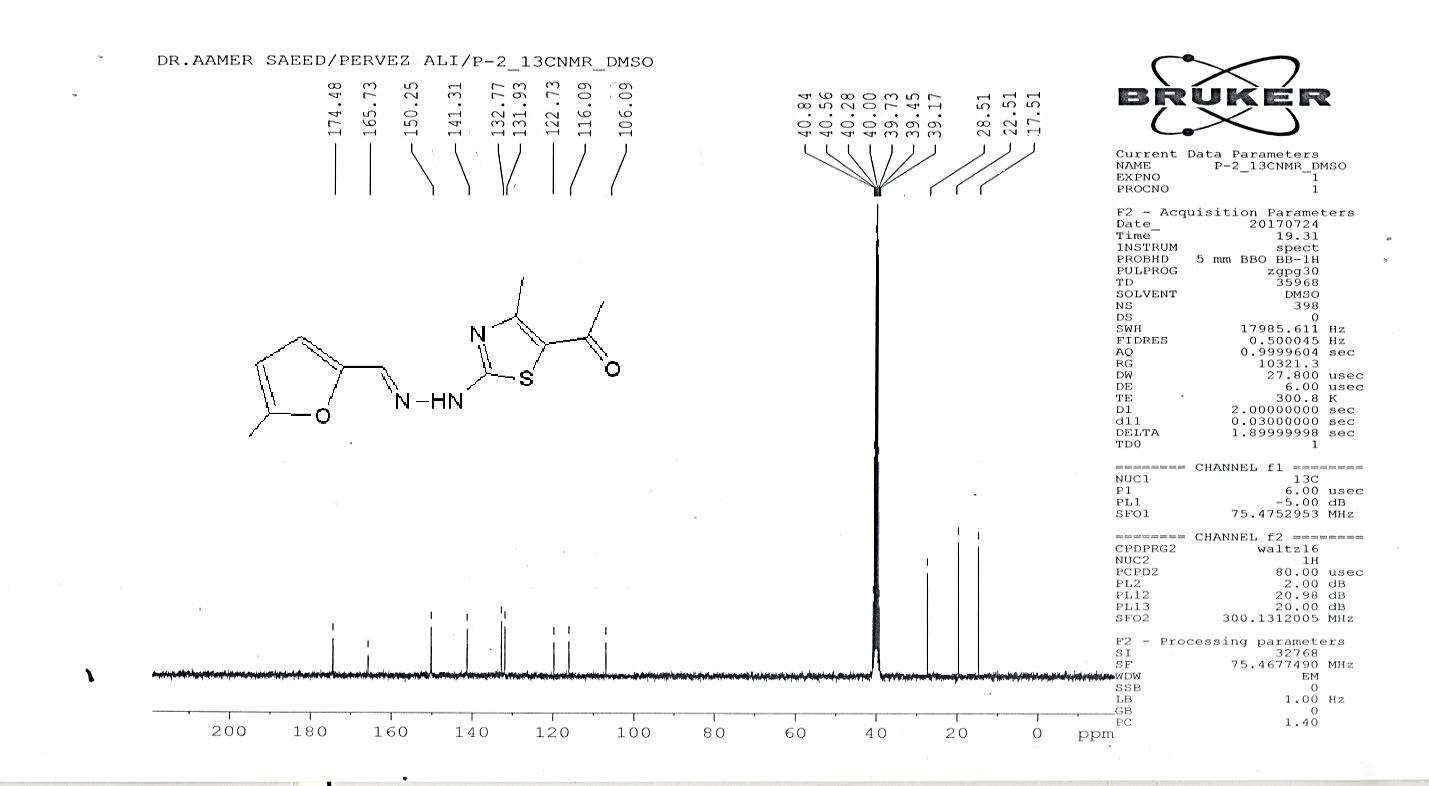
**

**Figure S8.** The ^13^CNMR spectra of *(E)-*1-(4-methyl-2-(2-((5-methylfuran-2-yl) methylene) hydrazinyl)thiazol-5-yl)ethanone

**
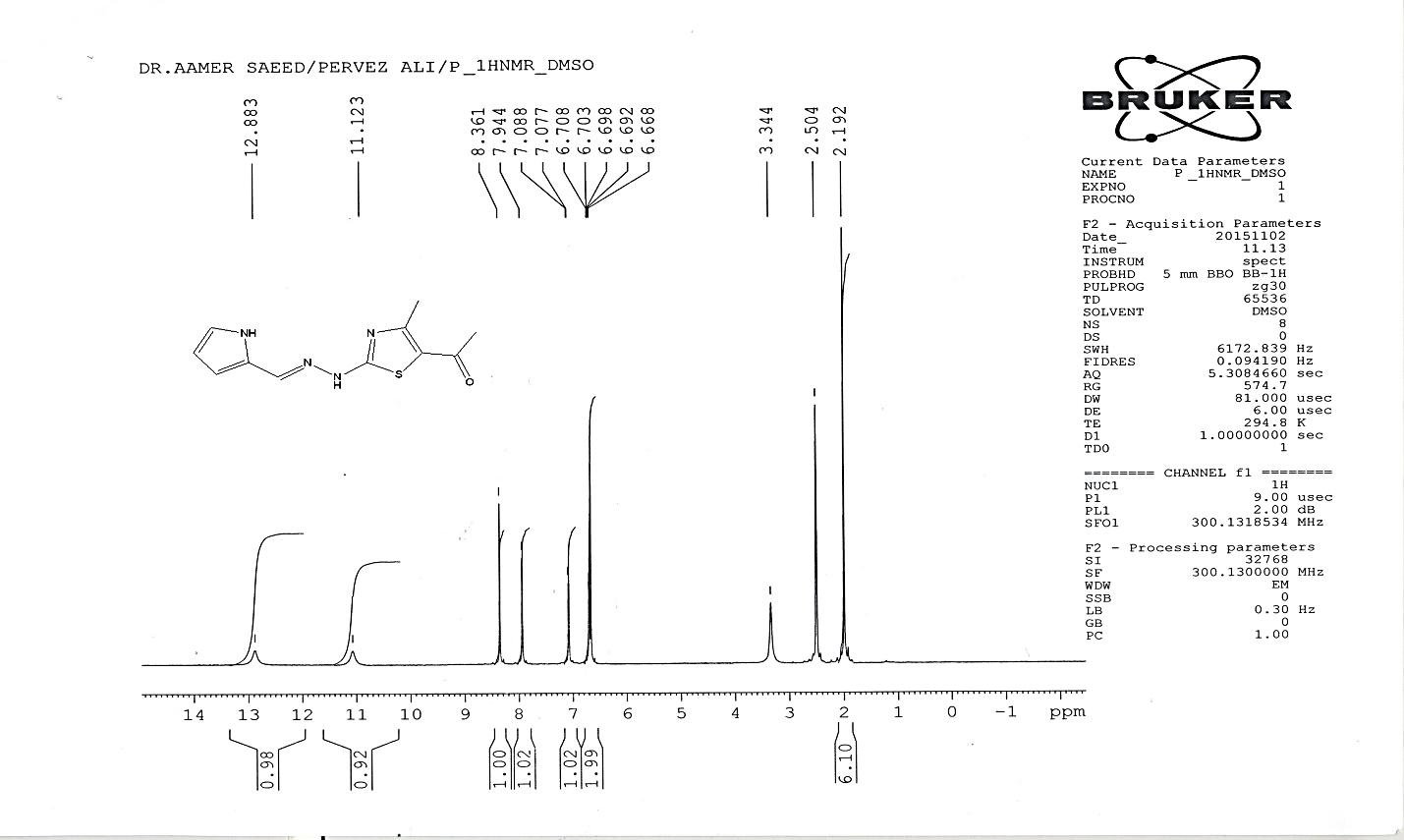
**

**Figure S9.** The ^1^HNMR spectra of *(E)-*1-(2-(2-((1H-pyrrol-2-yl)methylene)hydrazinyl)-4-methylthiazol-5-yl)ethanone

**
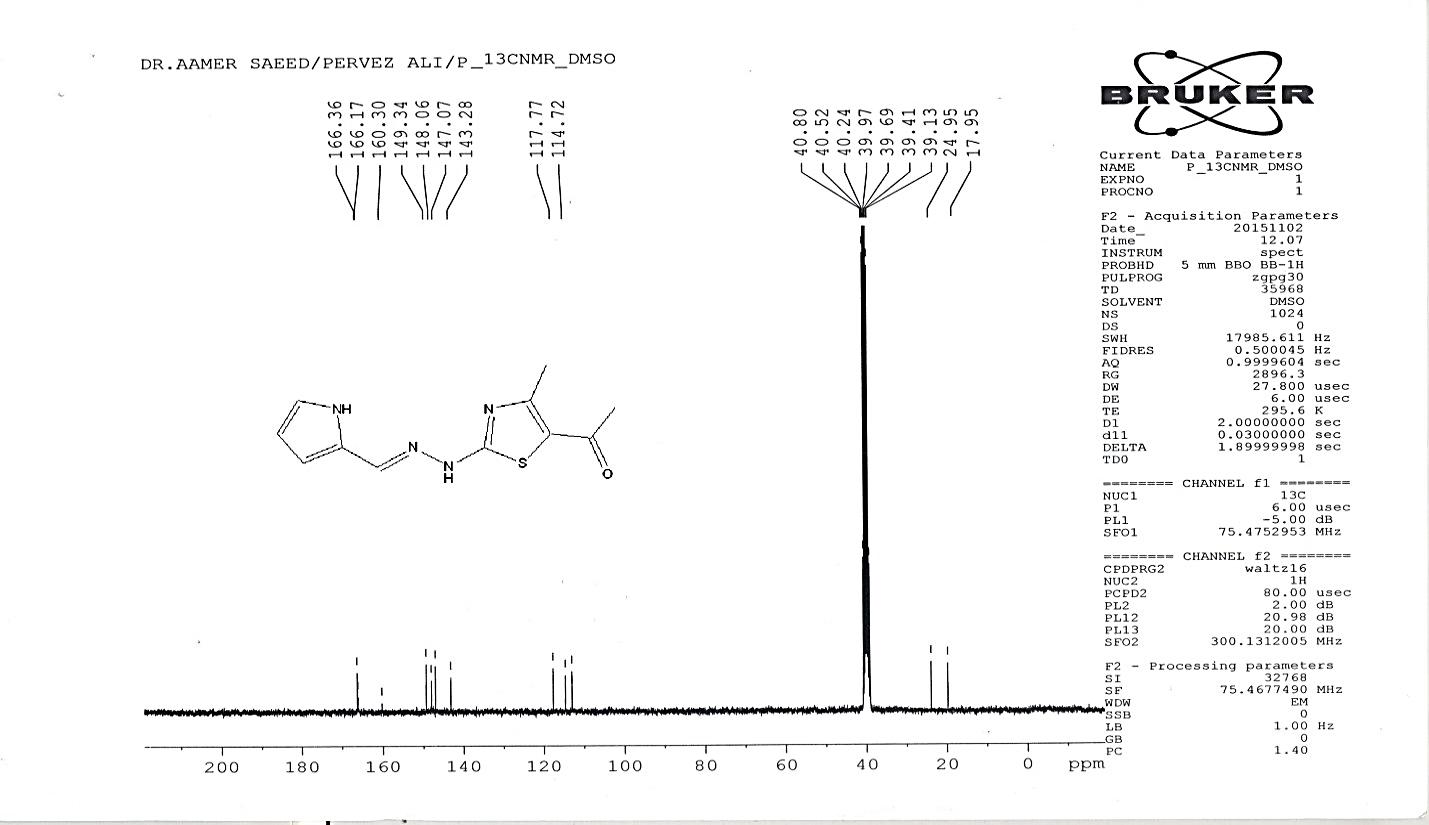
**

**Figure S10.** The ^13^CNMR spectra of *(E)-*1-(2-(2-((1H-pyrrol-2-yl)methylene)hydrazinyl)-4-methylthiazol-5-yl)ethanone


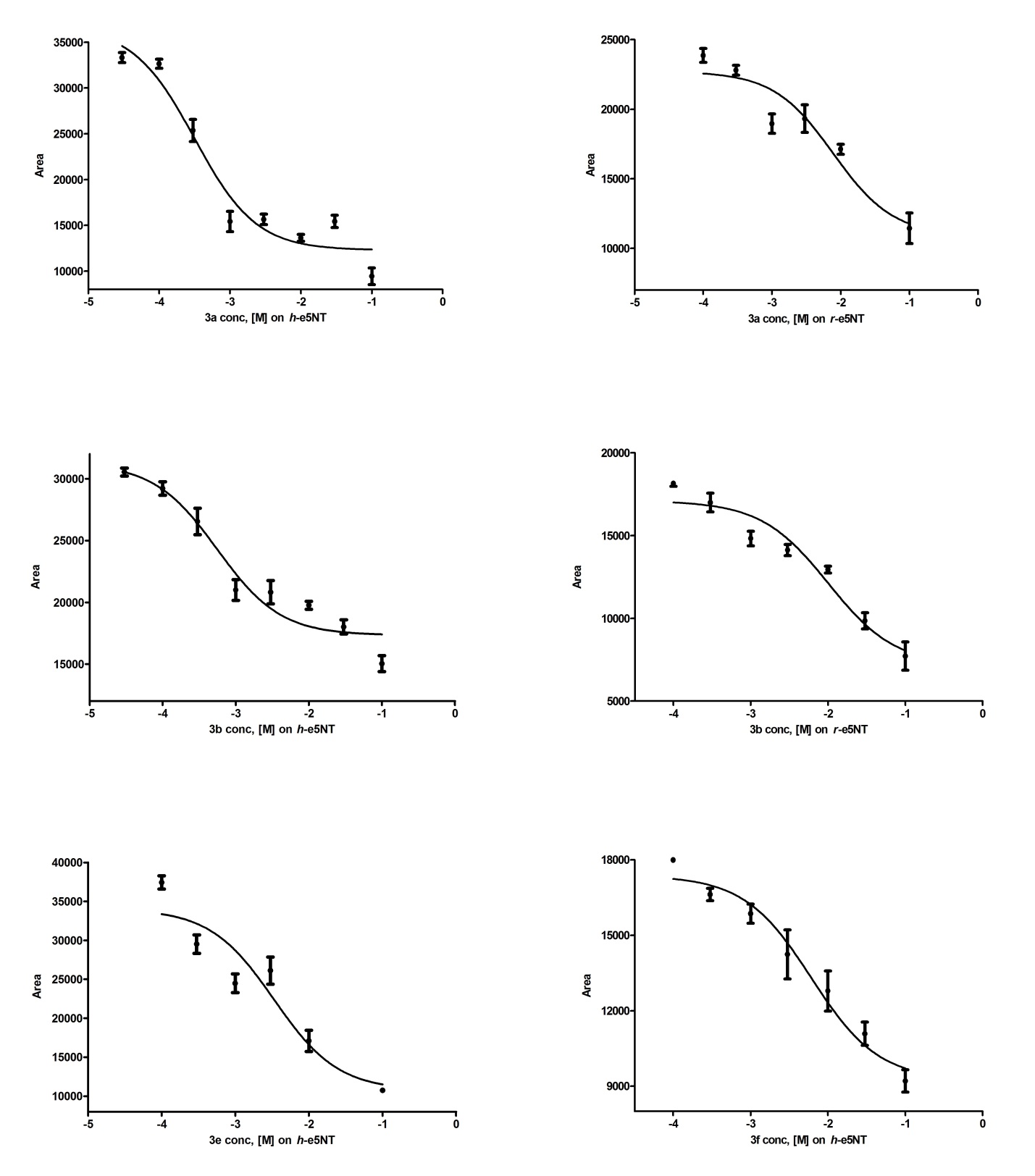


**Figure S11.** The IC_50_ data of test compounds on *h-*e5**ʹ**NT and *r-*e5**ʹ**NT enzymes.

The IC_50_ of 3a was 0.32 ± 0.03 on *h-*e5**ʹ**NT and 7.81 ± 0.89 on *r-*e5**ʹ**NT

The IC_50_ of 3b was 0.56 ± 0.07 on *h-*e5**ʹ**NT and 10.1 ± 0.58 on *r-*e5**ʹ**NT

The IC_50_ of 3e was 3.36 ± 0.12 on *h-*e5**ʹ**NT

The IC_50_ of 3f was 6.19 ± 0.32 on *h-*e5**ʹ**NT

1. [↑](#footnote-ref-1)
